# Bay watch: Using unmanned aerial vehicles (UAV’s) to survey the box jellyfish *Chironex fleckeri*

**DOI:** 10.1371/journal.pone.0241410

**Published:** 2020-10-29

**Authors:** Olivia C. Rowley, Robert L. Courtney, Sally A. Browning, Jamie E. Seymour

**Affiliations:** Australian Institute of Tropical Health and Medicine, James Cook University, Cairns, Queensland, Australia; Universita degli Studi di Genova, ITALY

## Abstract

Biological investigations on free ranging marine species are regarded as challenging throughout the scientific community. This is particularly true for ‘logistically difficult species’ where their cryptic natures, low abundance, patchy distributions and difficult and/or dangerous sampling environments, make traditional surveys near impossible. What results is a lack of ecological knowledge on such marine species. However, advances in UAV technology holds potential for overcoming these logistical difficulties and filling this knowledge gap. Our research focused on one such logistically difficult species, the Australian box Jellyfish (*Chironex fleckeri*), and we investigated the capacity of consumer grade UAV technology to detect this, highly venomous, target species in the inshore waters of Northern Queensland Australia. At two sites in the Weipa area, we utilized video analysis, visual count comparisons with a netted animal tally, and evaluated the role of associated environmental conditions, such as wind speed, water visibility and cloud cover on jellyfish detection rates. In total fifteen, 70 meter transects were completed between two sites, with 107 individuals captured. Drone success varied between the two sites with a significant difference between field and post-field (laboratory) counts. Animal size and cloud cover also had significant effects on detection rates with an increase in cloud cover and animal size enhancing detection probability. This study provides evidence to suggest drone surveys overcome obstacles that traditional surveys can’t, with respect to species deemed logistically difficult and open scope for further ecological investigations on such species.

## Introduction

Biological investigations on free ranging marine species are widely regarded as challenging throughout the scientific community. This is especially true for marine species that are considered logistically difficult, in that they often; occur in low abundance, are patchy in distribution, are difficult to visually detect and identify, and/or occur in environments that make them difficult and/or dangerous to sample. For these ‘logistically difficult species’ employing traditional survey techniques, such as visual monitoring and population counts, is often a costly endeavour with a very low rate of success and, in some instances, is known to generate poor quality data [1]. As a result, survey endeavours for these species are regarded as near impossible and often not undertaken [1]. Consequently, key ecological knowledge on these animals, such as distribution and abundance estimates alongside seasonality trends, are very limited.

Recent advances in remote sensing technology offer the potential to improve field surveys for logistically difficult species in particular, advances made on unmanned aerial vehicles (UAV’s) or drones [2, 3]. Traditionally, drones were restricted to those that could obtain difficult aircraft permits and meet high budgetary requirements but recent developments with respect to licencing, legislation and in the production of smaller consumer grade drones, mean that these tools are now more affordable, transportable and simpler to use than ever before [2]. This ‘off the shelf’ accessible nature, alongside the improved quality of onboard remote sensing instruments, has resulted in the emergence of an innovative and exciting era for wildlife research.

There are now ever increasing instances of drones being successfully used for monitoring free ranging marine vertebrates with a dominance in the literature towards large iconic megafauna, such as whales [4–6], sharks [7–10] dolphins [11, 12] sea turtles [13–15] and rays [9, 16]. These studies have highlighted the capacity of UAV monitoring to outperform manual boat-based surveys not only in terms of data quality but also cost and efficiency [17–19]. However, synonymous to traditional sampling strategies, UAV survey success is fundamentally linked to environmental conditions and technological limitations, with variables such as contrast, sun angle and water turbidity, alongside battery life and camera resolution, heavily effecting overall outcomes [15, 20]. Regardless, the prevalence and success of marine UAV faunal studies is widely acknowledged with some believing the applications for ecology is limitless. Yet to date research neglects to consider, or quantify, the significant role drones could play in monitoring smaller cryptic marine organisms; such as cnidarians, particularly in situations where traditional survey strategies are considered to have low rates of success, or which involve high levels of danger and risk.

Jellyfish are gaining increased attention worldwide due to the devastating effects of blooms on local ecosystems and, for some species, medical and economic implications due to their painful and potentially fatal stings. In areas around the world dramatic increases in the number and density of scyphozoan species has resulted in fishery collapses and caused significant losses to aquaculture stocks [21, 22]. Cubozoans, a family of box shaped cnidarians which often occur in less dense aggregations but are known for their highly venomous sting, have caused millions of dollars in lost tourist revenue in several different geographical locations such as Waikiki beach Hawaii, Queensland Australia and the Mediterranean coast [23–25]. However, despite these implications, there is a lack of data on distribution, density and spatial and temporal patterns for these marine invertebrates. This absence has been directly related to problems with sampling and monitoring, as these organisms are not only difficult to visually detect but their physical nature make them inherently problematic to sample [26, 27].

Despite these complications, a limited number of studies have demonstrated the ability of drones to obtain high quality, large area, static imagery of scyphozoan jellyfish and, through automated image processing software, accurately estimate the size and density of these aggregations [19, 28]. However, not only has this literature solely focused on the more common scyphozoan species, but the quantification methods rely heavily on slow moving mass aggregations (>200 individuals) for image targeting, alongside contrast (i.e. light animals on dark backgrounds), and consistencies in shape and colour, for target detection. Thus, for the more logistically difficult species, such as cubozoan jellyfish, which are known for their low individual numbers, cryptic transparent colouration and active nature, cross context applicability of these detection and quantification methods are unknown.

The aim of this project was to investigate the capacity of consumer grade UAV technology to detect a logistically difficult, and highly venomous, species of cubozoan jellyfish, *Chironex fleckeri*, in the inshore waters of northern Queensland Australia. To quantify the success and applicability of the UAV survey method, aerial footage obtained via drone transects were analysed for the visual detection of *Chironex fleckeri*, which were then directly compared to ground truthed counts using traditional netting techniques. The analytical factors that influence the detectability of this species, via drone surveys, were established and the influence of environmental variables, such as wind speed, turbidity and cloud cover on the detectability of this species was determined.

## Methods

### Target species

*Chironex fleckeri*, commonly known as the Australian box jellyfish, is a multi-tentacled jellyfish in the class Cubozoa. Characterised by its large size (>30cm inter-pedalial distance), transparent colouration, active nature and highly venomous sting, these animals occur in the inshore waters of Northern Australia seasonally from September to June each year. The Australian box jellyfish is responsible for over 60 deaths in Australian waters over the last 70 years [29]. As a result, this species severely influences the way water activities are undertaken in the areas where these jellyfish are present, subsequently, leading to a significant cost to Australian tourism.

All animals were collected in accordance with permit number 204653 (Department of Agriculture and Fisheries, Queensland, Australia).

### Study site and field sampling technique

**Fig 1:**
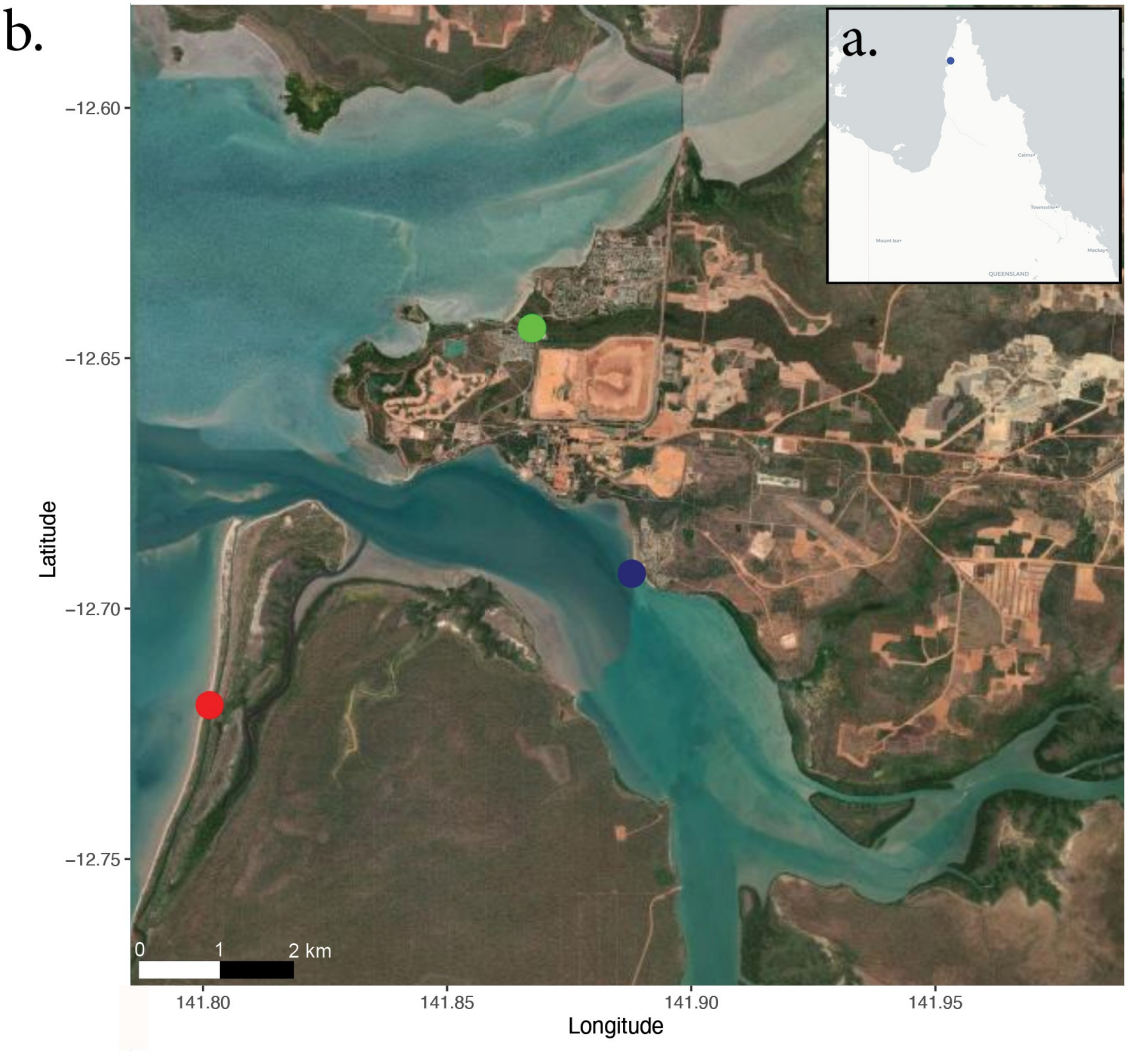
Map of Weipa area sample locations in far north QLD Australia. a- Map of Far North Queensland (Weipa marked in blue) b—Sample sites, Weipa township (green), Westminster (red) and Napranum (blue). Satellite maps were generated using the leaflet package in R (base map data from OpenStreetMap and OpenStreetMap Foundation under the open database licence, licenced as CC BY-SA 2.0).

**Fig 2:**
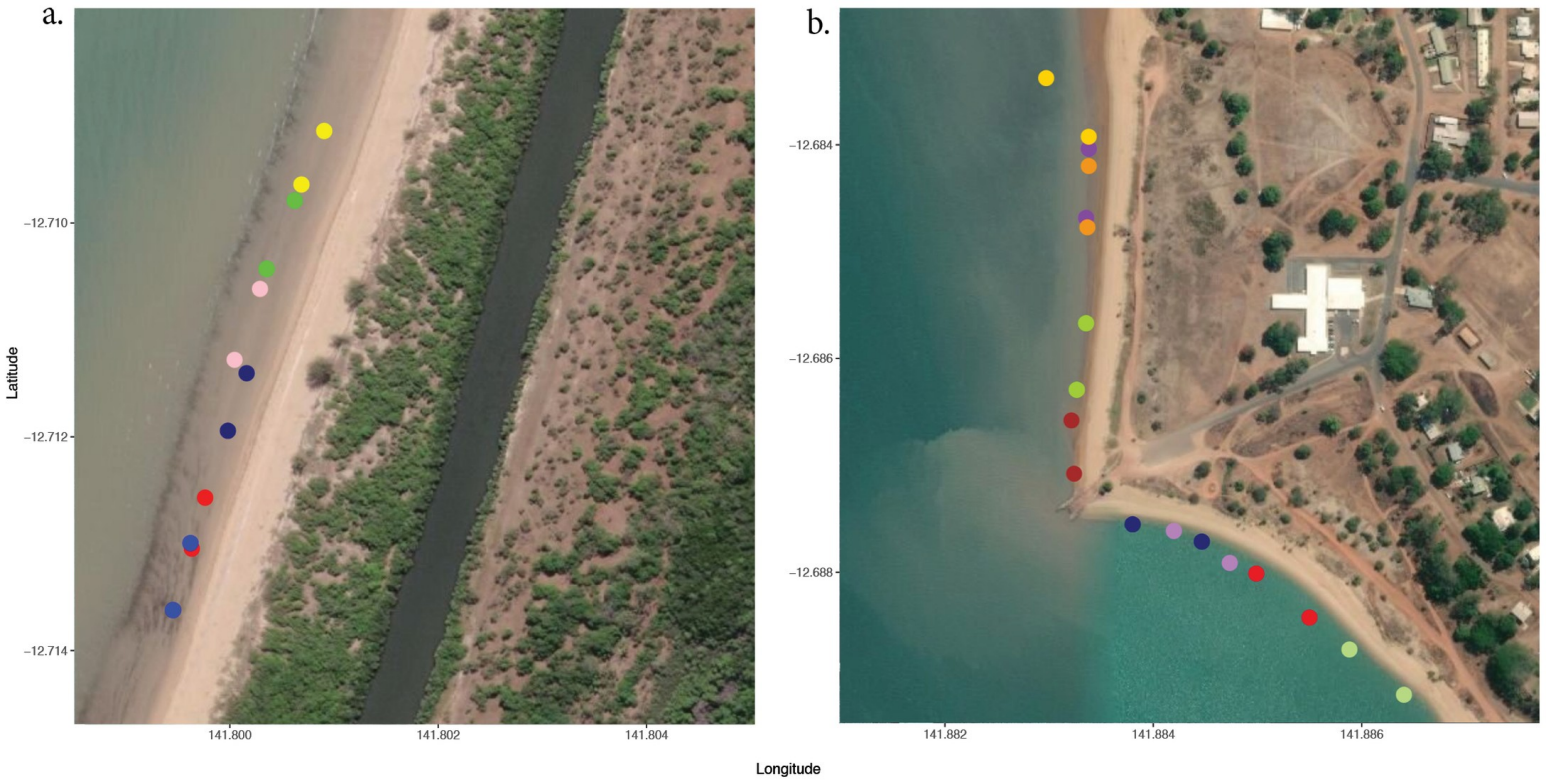
Transect distributions at sample locations. Westminster (a) and Napranum (b). Each pair of coloured dots represent the start and finish of transect. Dots overlapping were completed on sepperate days. Satellite maps were generated using the leaflet package in R (base map data from OpenStreetMap and OpenStreetMap Foundation under the open database licence, licenced as CC BY-SA 2.0).

Field sampling occurred at two geographical locations near Weipa, Queensland Australia, Westminster (12°698839’S,141°802622’E) and Napranum (12°686740’S, 141°885706’E) (Fig 1). Both locations were sampled between November 26 and December 1, 2019. Geographical locations were chosen for animal specificity, with *Chironex fleckeri* reliably occurring at both locations, and also to ensure variability in background complexity and water conditions. Each location was subsampled with multiple sites per transect with between 7–8 transects completed at each site’s dependant on weather conditions. Transects were, on average, 70m long by 6m wide but varied at each location due to beach shape and water conditions. In total, flight paths covered between 1750-2450m^2^ of inshore environment at both sites over the course of six days. Transects were spaced out geographically and transects that are located next to each other were sampled on alternating days (Fig 2). All sampling efforts occurred in the morning between the hours of 8–11am to give the highest probability of catching animals, high morning tides, and to remove any confounding effects that may result from altering the time of day for sampling.

Each transect involved two components; netting and drone, which occurred simultaneously. A 70m seine net, with a 2.5m drop, was fed off the back of a 5.8m research vessel. A mesh size of 25mm reduced nearly all animal bi catch. The net was deployed ~6m parallel to the shore and anchored at each end while the drone transect was run. Following net placement, a researcher was situated between the shore and net to minimise animal loss. Upon completion of each transect both anchors were released and simultaneously each corner was slowly pulled towards the beach. Animals caught in the netted area were retrieved by hand and placed on the beach for tallying and size processing. Medusa size measurements were taken as the inter-pedalia distance (IPD) which is the distance between the base of two adjacent pedalia. The net functioned as a ‘hard count’ enabling the capture of all jellyfish in the survey area while IPD data enabled assessment of drone effectiveness relative to target size.

Before net retrieval, a drone was flown at two different altitudes from the water’s surface (6 and 10m). Drone altitude was selected on the basis of reducing prop wash (a phenomena where turbulent air from the propellers causes rippling of water), enabling a suitable survey area, ensuring target animals would be of a detectable size (on average taking up approximately between 6–7% of the total frame) and retaining pixel resolution. The drone flew in the centre of each transect, parallel to the research vessel, at ~0.4m/s to match the speed at which the net was deployed. A ‘live count’ of jellyfish seen was taken by the pilot during each transect and recorded. Each transect was run six times in the same direction (three times at each height) and a single transect, for each height, was selected by taking into account factors such as wake wash, glare and wind gusts.

### Aircraft configuration and operation

All flights were completed using a DJI Mavic 2 zoom drone. The inbuilt 12 mega pixel camera was equipped with a ND16 lenses polariser and video was recorded at a 70degree angle to reduce sea surface glare which, synonymous to other similar studies, optimised footage for animal detection [8]. During each transect the camera was set to record the entirety of the flight, with footage in full high definition (4k) at 30 frames s^-1^ in the widest field of view setting. A singular battery was used for each net haul, giving the drone approximately 20 minutes of flight time (enough to orient drone, complete both 10 and 6m transects and return home with about 10% battery remaining). The drone was controlled with a DJI Mavic remote and camera feed was viewed, by the pilot, in real time with DJI ‘first person’ googles (1080p HD screen). The use of goggles also ensured there was no glare on the telemetry screen and to reduce lag between drone and remote screen. In accordance with Australian Civil Aviation Safety Authority (CASA) regulations all flights were completed within the pilot’s line-of-sight (when not wearing goggles) and, in cases where line of site could not be maintained by the pilot, a secondary observer had visual contact with the drone at all times.

### Flights

In total approximately 450 minutes of footage was recorded over 15 transects. All drone flights were completed by the same pilot and GPS reference points were taken at the start and end of each flight using the inbuilt drone GPS and cross-referenced with the GPS from the research vessel. For each of the two sites the direction of all flights remained the same; Westminster—NE to SW, Napranum- NW-SE. Direction was selected on the basis of beach orientation and the reduction of sea surface glare.

### Environmental data

Following the completion of each transect eight environmental variables were recorded. These included: average and maximum wind speed (km h^-1^) and wind direction (decimal degrees) (measured using a handheld windmeter), water visibility (m obtained via a 30 cm Secchi disk), cloud cover (scored from 0–8 representing no cloud cover and 100% cloud cover respectively), and sea temperature (°C) and depth (m) (measured via onboard Lowrance fish finder and thermometer). Alongside this tide direction (at time of sampling) and tidal peak was also noted.

### Drone footage review

One month was left between field analysis and *post-hoc* analysis and all transects were triplicated and given dummy numbers to reduce observer bias by eliminating any indicators of date, day or flight number. Due to the biology and high activity levels of target species, common image processing techniques, such as background removal or thresholding, was deemed unsuitable and video footage of each transect was reviewed on an 11inch MacBook pro with a retina display. Each transect produced between 2–3 minutes of footage (dependent on wind conditions) and analysed in real time. All footage was reviewed by two individuals, the drone pilot and an independent observer, to check for bias with the drone pilot. For validation, the observers noted the time point at which each animal was identified and where, in the field of view, the observation was made.

### Statistical analysis

Observer effect and technique was analysed using a linear mixed model which allowed net haul number to be factored as a unit of ‘within group’ replication. For location-based analysis a simple ANOVA was used. Generalised linear mixed model (GLMM) with a binomial distribution and a logit link function was used to assess the effect of various factors on the overall jellyfish detection rate of the UAV. The response variable, drone effectiveness, was classified as detection parameter calculated via the number of jellyfish detected vs the total number undetected and was analysed against the above-mentioned environmental parameters alongside other factors such as altitude and animal size. Final model selection was made on the basis of Akaike information criterion (AIC). All statistical analyses and mapping were carried out using R 3.2.1 software (R core team).

## Results

In total fifteen, 70 meter, transects were conducted over an eight-day period in the Weipa area. Of these, six transects were completed at Westminster (site 1) and nine at Napranum (site 2). All transects were completed in inshore environments with water depth never exceeding 2m. *Chironex fleckeri* was present in all but one net haul. In total, 103 jellyfish were captured with an average of seven individuals per net haul. As, within each net haul, each jellyfish had two chances of being detected, once on 7.5m passover and another at 9.5m, there was a total of 206 possible jellyfish detections thorough this study.

### Animal size and count by location

**Fig 3:**
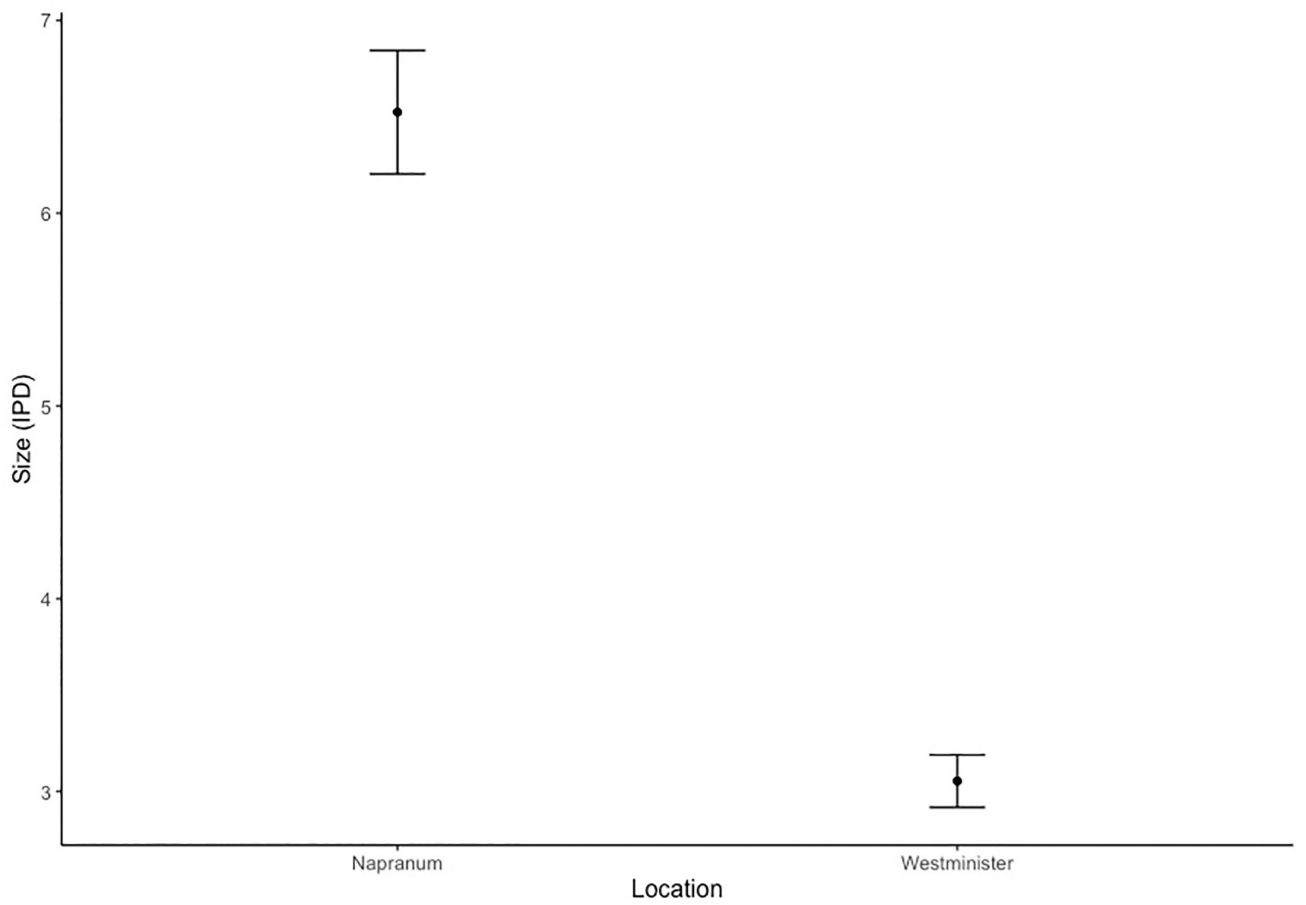
Animal size by geographic location. Size is a measure of inter-pedalia distance (IPD) and points represent group means (±SE) and have been grouped by location.

**Fig 4:**
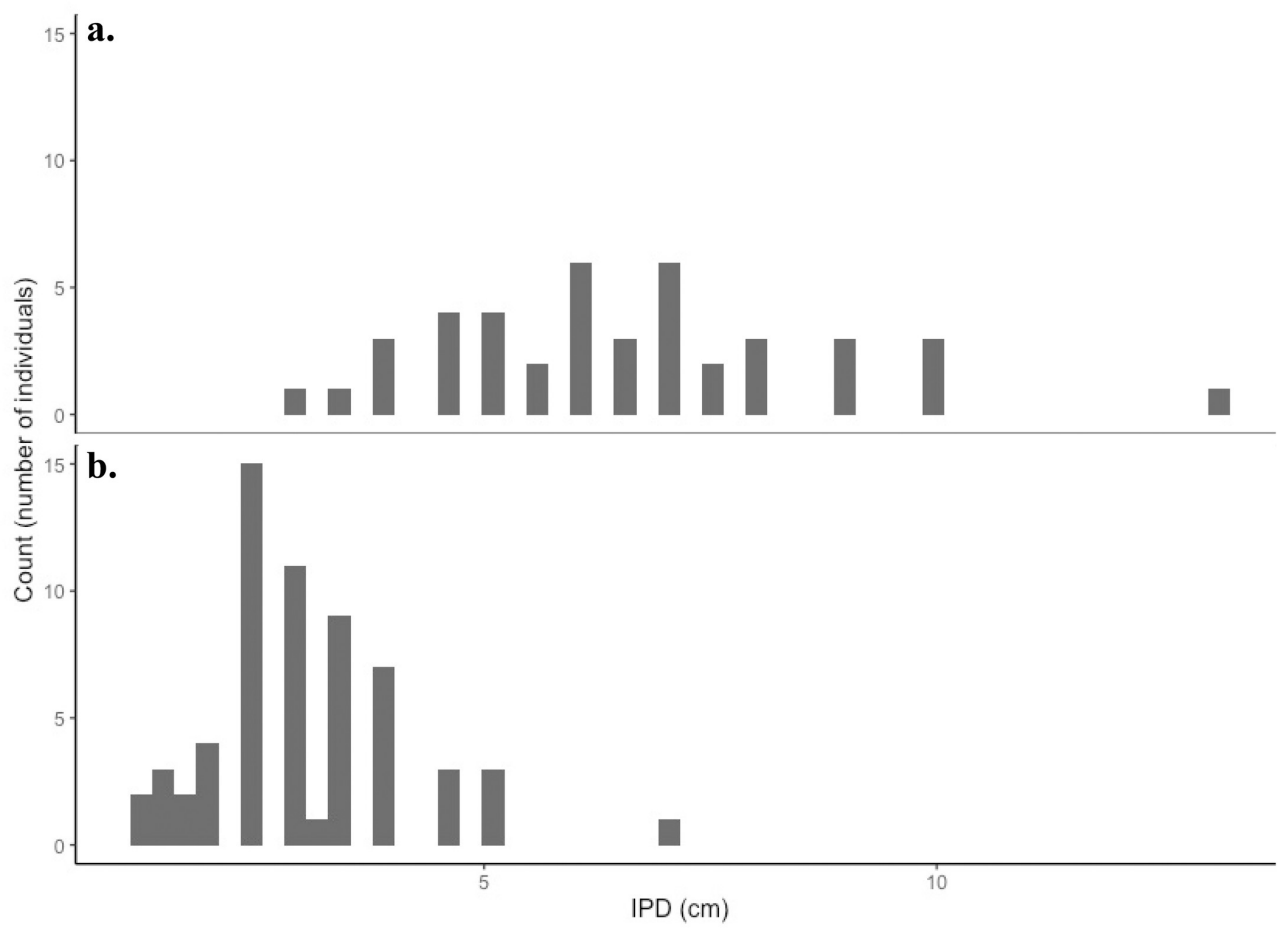
Animal size and catch frequency by geographic location. The bell size was measured as inter-pedalia distance (IPD in cm), and the individuals have been grouped by location, Napranum (a) and Westminster (b).

Animal sizes varied between locations and transects. Animals captured at Westminster were, on average, significantly smaller (F_1,100_,123.9, P <0.001) than those caught at Napranum (Figs 3 and 4). The average IPD of animals by site were 3.05cm (7.5m ~ 5.856 pixels, 9m–~ 4.514 pixels) and 6.23cm (7.5m–~11.962 pixels 9m–~ 9.220 pixels) at Westminster and Napranum respectively. A small, site-specific, disparity existed with regards to the average number of individuals in a net haul, with hauls at Westminster returning on average nine netted individuals as opposed to six at Napranum, but this was found to be statistically insignificant (F_1,12_, = 1.573, P>0.001). Napranum, was the only site to return an empty net haul.

### Detection method and detectability by location

**Fig 5:**
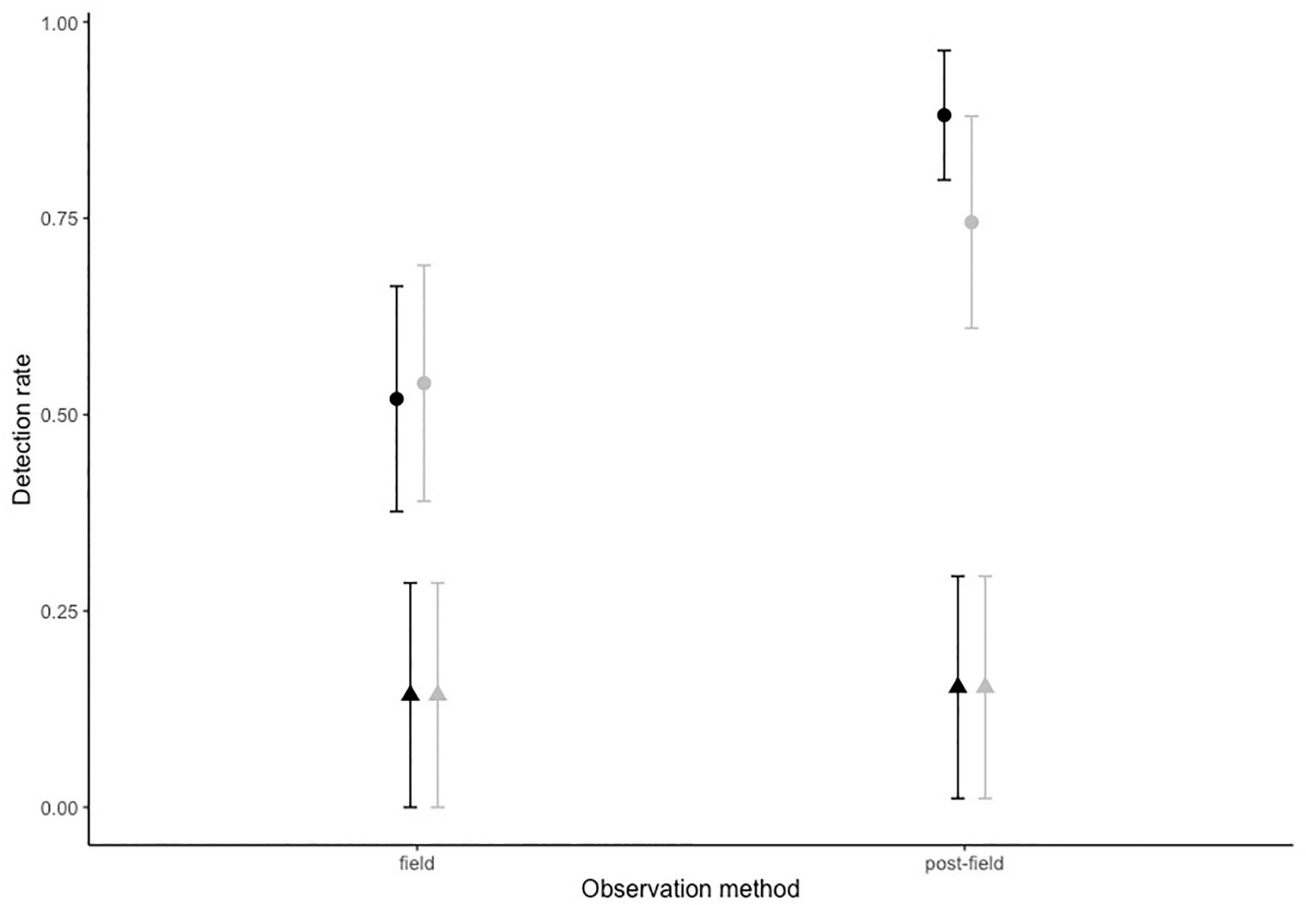
Detection rate by method and location. Field observations were made live by the drone pilot and post-field observation are defined as the maximum detection rate between lab observers. All sites are shown, Westminster (triangle), Napranum (circle). All detection rate is a decimal percent shown are the group means and SE. Groups are coloured by observation altitude 7.5m (black), 9.5m (grey).

Observation method had a significant effect on drone detection success, but this was dependent on site (Fig 5 and Table 1). Of the total, 206, jellyfish detection opportunities, only 12% (25 counted individuals) were made by the drone pilot in the field as opposed to 31.6% (or 65 individuals) in post-field observations. Analysis, on post-field jellyfish detection rates, revealed no statistical difference between observers (pilot vs independent) regardless of site (F_1,43_ = 0.3114, P>0.001), so post-field observation data was taken as the maximum detection rate of the two observers. Detection success, regardless of method, was very small (between two and three percent) at Westminster with only four detections made out of a possible 122 (3%). However, post-field observations, made by the pilot and independent observer at Napranum, had a significantly higher rate of jellyfish detection (76% total, maximum 100%—minimum 38%) than the pilots ‘in field’ observations (28% total, maximum 100%—minimum 0%) (F_1,19_ = 7.3892, P = 0.01324).

**Table 1:** Summary of haul and transect data and associated detection rates.

Haul number	Location	Animal Talley (individuals)	Animal IPD (cm—mode)	Altitude (m)	Field Count (individuals)	Video Analysis (individuals)		Video Analysis (Maximum detection rate)
						Observer one (pilot)	Observer two (Independent)	
1	Westminster	14	2.25	7.5	**0 (0%)**	1 (7%)	1 (7%)	**1 (7%)**
				9.5	**0 (0%)**	0 (0%)	1 (7%)	**1 (7%)**
2	Westminster	9	3.5	7.5	**0 (0%)**	0 (0%)	0 (0%)	**0 (0%)**
				9.5	**0 (0%)**	0 (0%)	0 (0%)	**0 (0%)**
3	Westminster	15	2.8	7.5	**0 (0%)**	0 (0%)	0 (0%)	**0 (0%)**
				9.5	**0 (0%)**	0 (0%)	0 (0%)	**0 (0%)**
4	Westminster	3	3	7.5	**0 (0%)**	0 (0%)	0 (0%)	**0 (0%)**
				9.5	**0 (0%)**	0 (0%)	0 (0%)	**0 (0%)**
5	Westminster	3	2.5	7.5	**0 (0%)**	0 (0%)	0 (0%)	**0 (0%)**
				9.5	**0 (0%)**	0 (0%)	0 (0%)	**0 (0%)**
6	Westminster	17	2.5	7.5	**0 (0%)**	0 (0%)	0 (0%)	**0 (0%)**
				9.5	**0 (0%)**	0 (0%)	0 (0%)	**0 (0%)**
7	Westminster	1	9	7.5	**1 (100%)**	1 (100%)	1 (100%)	**1 (100%)**
				9.5	**1 (100%)**	1 (100%)	1 (100%)	**1 (100%)**
8	Napranum	6	6	7.5	**2 (33%)**	6 (100%)	6 (100%)	**6 (100%)**
				9.5	**4 (67%)**	6 (100%)	6 (100%)	**6 (100%)**
9	Napranum	12	7	7.5	**2 (17%)**	12 (100%)	7 (58%)	**12 (100%)**
				9.5	**3 (25%)**	12 (100%)	8 (67%)	**12 (100%)**
10	Napranum	13	5	7.5	**1 (8%)**	5 (38%)	5 (38%)	**5 (38%)**
				9.5	**2 (15%)**	6 (46%)	4 (31%)	**6 (46%)**
11	Napranum	2	7	7.5	**2 (100%)**	2 (100%)	1 (50%)	**2 (100%)**
				9.5	**2 (100%)**	0 (0%)	0 (0%)	**0 (0%)**
12	Napranum	1	6	7.5	**1 (100%)**	1 (100%)	1 (100%)	**1 (100%)**
				9.5	**1 (100%)**	1 (100%)	0 (0%)	**1 (100%)**
13	Napranum	4	7	7.5	**1 (25%)**	4 (100%)	3 (75%)	**4 (100%)**
				9.5	**1 (25%)**	2 (50%)	2 (50%)	**2 (50%)**
14	Napranum	3	10	7.5	**1 (33%)**	2 (67%)	2 (67%)	**2 (67%)**
				9.5	**0 (0%)**	3 (100%)	0 (0%)	**3 (100%)**
15	Napranum	0	0	7.5	**0 (100%)**	0 (100%)	0(100%)	**0(100%)**
				9.5	**0 (100%)**	0 (100%)	0(100%)	**0(100%)**
Total	Westminster	62	3.05	7.5	**1 (14%)**	2 (15%)	2 (15%)	**2 (15%)**
				9.5	**1 (14%)**	1 (14%)	2 (15%)	**2 (15%)**
	Napranum	41	6.52	7.5	**10 (24%)**	32 (78%)	25 (60%)	**32 (78%)**
				9.5	**11 (27%)**	30 (73%)	20 (49%)	**30 (73%)**

### Altitude

Flight altitude had no statistical effect on jellyfish detection success (see Table 2, P >0.001) (Fig 5).

**Table 2:** Summary of generalised linear model for the effect of environmental variables, site and altitude on jellyfish detection probability.

Coefficients	Estimate	Std. error	Z value	P-value
Intercept	-9.7077	8.2840	-1.172	0.24125
Cloud cover	4.2355	1.6430	2.578	<0.001
Wind speed	0.6222	0.8972	0.694	0.48795
Visibility	1.6431	3.8801	0.423	0.67196
Size (mode)	0.9988	0.3785	2.639	<0.001
Altitude	-0.1467	0.2717	-0.540	0.58931
Site	-1.8920	3.2135	-0.589	0.55602

### Drivers of detection—GLM results

All environmental conditions were considered mild thorough the duration of this study. Wind conditions varied in intensity between 2 and 4.5m/s between flights and gusts of up to 7m/s. Cloud cover ranged from 0 (clear sky’s) to 1 oktas (full cloud cover) and the range of water visibility was small (scoring between 1 and 1.75m between days).

**Fig 6:**
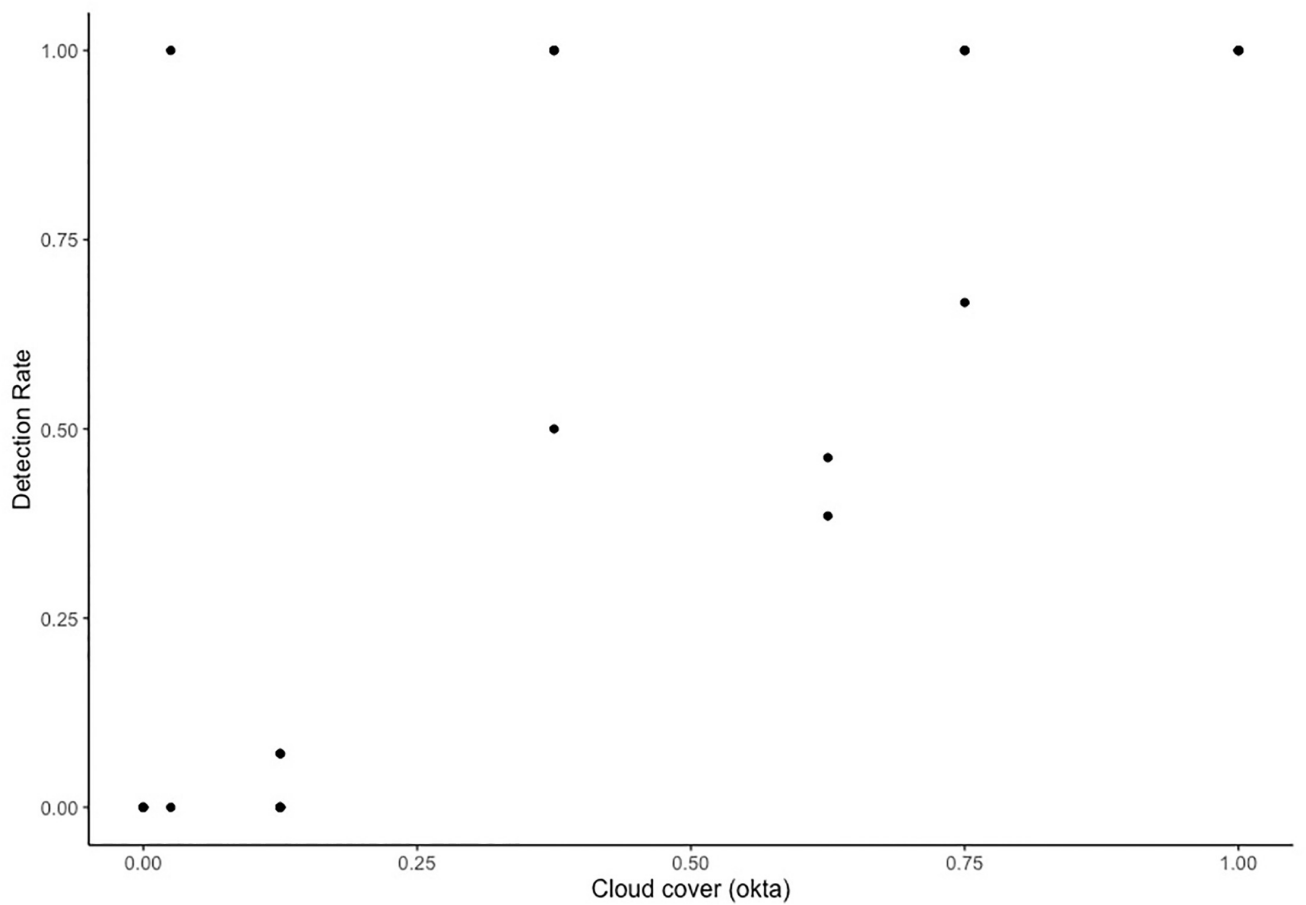
Cloud cover vs jellyfish detection rate. All detection data is max detection rate from post-field detection (decimal percent) both altitudes have been pooled.

**Fig 7:**
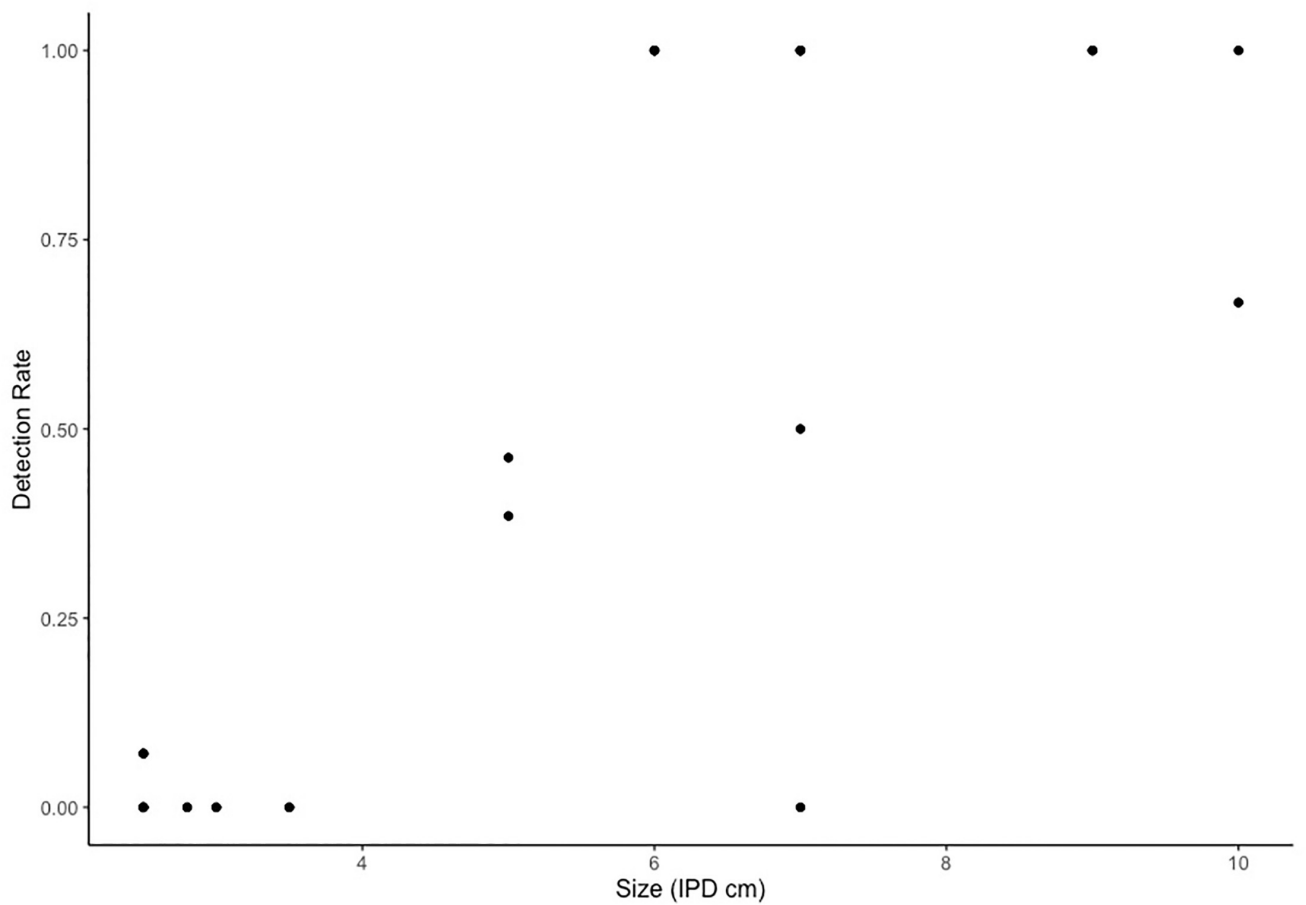
Plot of drone detection success by animal IPD (cm). All detection data is max detection rate from post-field detection (decimal percent) both altitudes have been pooled.

Cloud cover and animal size significantly influenced the probability of animal detection (P<0.001). Cloud cover was positively corelated with detection success post-field as an increase in cloud cover was found to improve levels of animal detection (Fig 6). There were three instances of 100% detection success with cloud cover lower than 0.50 oktas, however, these detections involved a very small number (one or two) of large animals. Likewise, an increase in animal size (mode IPD cm per transect) increased the probability of detection success (P <0.001 Fig 7). All remaining environmental variables tested (wind direction, sea temperature, wind speed and visibility) were statistically insignificant and the final model (Table 2) selection was made on the basis of AIC.

## Discussion

Our data suggests that using small, consumer-grade drones hold significant promise as an alternate method for detecting *Chironex fleckeri* in inshore shallow-water environments. Specifically, we highlight the significant role of observation technique, target size, environmental conditions and logistical factors, such as flight altitude, on detection probabilities. We emphasize and extrapolate these advantages, highlighting the scope and capacity of this survey technique for other species considered logistically difficult.

In this study, there was a two-and-a-half-fold difference in the number of animals detected by the lab observers when compared to that of the ‘in field’ drone pilot. This suggests that, for optimal detectability, drone footage should be collected in the field and analysed post-hoc. A large number of studies have reported this phenomenon, with similar levels of discrepancy, and have accredited this trend to many different factors [8]. These range from (i) the challenge for drone pilots to split their attention between flying and animal detection to (ii) technical difficulties involving telemetry screen resolution and glare [8, 20]. However, unlike previous studies, all piloting for this project was carried out using first ‘person-view DJI goggles. This headset provides the pilot with a high-resolution real-time video feed and, as the headset is fully enclosed, eliminates any potential for telemetry screen glare. This interface screen is, however, significantly smaller than that of the 13inch lab computer. What this technique could not offer was a playback capacity that matches that of ‘post-field’ analysis. The use of playback, to slow down, pause, or replay portions of transects was used post analysis and offers an unmatched opportunity to validate signings that cannot be met in the field. This, alongside the capacity of the drone pilot to split attention, may have reduced the pilot’s performance resulting in the discrepancies between field and post-field animal detection rates.

There was a small difference between detection success at the two tested altitudes, but this was statistically insignificant. Target altitudes were set on the basis of reducing prop wash while also ensuring each 70m transect could be completed on a single pass, but as heights were only 2m apart further investigations are needed to fully explore this relationship.

Animal size had a significant effect on jellyfish detectability post-hoc, as net hauls and corresponding transects with a larger modal animal size, had higher rates of animal detection (Fig 7). This pattern, while present at both sites, was most pronounced at Napranum. Target size, in remote sensing studies, is directly correlated to the number of on-screen pixels an object occupies. Thus, a larger animal occupies more on-screen pixels and is easier to detect. In addition, and perhaps more importantly in the context of this study given the transparent nature of the target animal, size was linked to an alteration in morphological traits which effected target contrast. Across all net hauls larger individuals were noted to have significantly wider tentacles and markedly more opaque gonadal tissue in the bell. This increased the contrast between the target animal and its background potentially aiding in detection.

Flights were exposed to a range of environmental conditions with varying levels of significance on jellyfish detection rates. Cloud cover was positively related to animal detectability with higher levels of cloud increasing the detection rate of jellyfish. Sun glare is a major confounding factor for detectability in marine environments [8, 20]. This is due to water’s high capacity to reflect light which generates glare. Reflection or glare leads to a loss of contrast between background and target making individuals harder to distinguish and surveys difficult [20]. To reduce this issue, the drone camera was equipped with a circular polarising filter, and adjustments were made to flying angle and camera gimbal position. Nevertheless, despite these adjustments sun glare was still present and made footage processing difficult. On days of high cloud cover, there was a noticeable reduction in sea surface glare as cloud functioned as a softening box, dispersing light evenly throughout the area and, as a result, increased detection rate. Sea state parameters, such as visibility, wind speed and wind direction, had no effect on rates of animal detection. These findings, when considered alongside the significant body of literature supporting role these environmental factors on UAV survey success, seem somewhat counterintuitive [7, 8, 10, 20]. However, these results reflect core ecological characteristics unique to the model system. *Chironex fleckeri* occurs in very shallow inshore environments [30]. While water turbidity has been identified as having a significant impact on detection rates for deep water and/or diving marine animals, which have the capacity to ascend below the waters sightability threshold, when considered in the context of shallow water and/or nearshore surveys, the significance is less as animals are, more often than not, detectable from the seafloor to surface [7, 11, 31]. All transects in this study occurred between 0.5 and 2m and the variation in water visibility, over the duration of sampling, was between 1 and 1.7m. Thus, the sightability threshold was never reached making this environmental factor redundant. Likewise, there was no significant effect of windspeed, or direction on rates of jellyfish detection and this result can be attributed to a combination of the video stability on the Mavic Pro Zoom, the relatively low wind conditions and calm sea state over the duration of this study.

This investigation also identified a high level of variation in relation to the detectability of targets between sites with transects at Westminster returning a very low detection rate compared to those carried out at Napranum (Table 1). While this difference was deemed non-significant by the final model it deserves acknowledegement. Aside from the discrepancy explained by target size and cloud cover, with Napranum having significantly larger animals and higher levels of cloud cover (Fig 3 and S1 Fig), this difference could be a result of background complexity as there was a noticeable difference in the bedform between the two sites. The bedform at Westminster was mottled in colour and complex than the Napranum site and, due to the shape and location of the Westminster beach, incoming waves cause the sandy substrate to form a ripple like texture (S1 Video). This paired with the angular approach of the waves meant the background for image capture was mottled and busy. It is widely regarded that it is harder to detect a target on a complex or mottled background, due to the way it interrupts the perception of outlines and target contrast, but due to experimental design this could not be tested [3, 32]. Future research should endeavour to quantify background complexity and explore its effect on detection success.

Historically, all drone-based jellyfish surveys have relied on high quality static imagery, and automated image processing software, to detect and count targets generating robust population estimates [19, 28]. For logistically difficult jellyfish species such as *Chironex fleckeri*, which have a tendency towards smaller numbers, shallow water environments and are considered cryptic in colouration (making silhouettes nearly indistinguishable from background substrate–S2 Video), the cross-context applicability of thresholding, as quantification method, was questioned. Throughout all transects in this study movement was vital for every jellyfish detection. Simple alterations in animal behaviour, for example when animals use their motor skills to sustain their position in the water column when boat wakes passed rather than move within the sampling area, made detection very difficult. Therefore, while high quality aerial video footage can certainly detect jellyfish presence, given the significance of target movement for detection, we have reason to believe this would be difficult off static images alone.

## Conclusion

This study provides evidence to suggest drone surveys overcome obstacles, that traditional surveys can’t, with respect to the field identification of *Chironex fleckeri* and opens scope for beach safety and ecological understanding of this logistically difficult species. We have highlighted the role of environmental factors and morphological animal characteristics on the overall detection rates and, for optimal success with this target species, high levels of cloud cover will aid in detection and all video should be analysed post-field (this could be as simple as having a secondary observer in the field watching a live feed). Subsequently, as with all studies of its kind, we found the data obtained favoured the detection of larger individuals. In order to optimise this approach, future research would benefit from further enquiry into the role of altitude on detection rates, a comparison of this method to boat based visual surveys, and, should also endeavour to improve detection rates of smaller specimens. Also, while we believe video analysis is a good quantification method, the discrepancies in the data, between netted samples and drone footage, mean that there is a need for a revised approach if this method was to be used to obtain true population estimates. The use of Unmanned Aerial Vehicles for the monitoring of free ranging marine species, such as jellyfish, has opened a gateway for ecological understanding. It is hoped that wide scale application of these methods will see enhanced ecological understanding not only of jellyfish but logistically difficult species as a whole.

## Supporting information

S1 Fig Cloud cover by site. Napranum, Westminster (mean ±SE).
(TIF)

S1 Video Comparative transect background complexity between sites. Napranum (a) and Westminster (b), all footage captured from 7.5m altitude with a 70degree angle.
(MP4)

S2 Video Aerial footage of *Chironex fleckeri*. a- Typical collection of *C*.*fleckeri* involves researchers scanning beaches and entering the water when a target is spotted, but target identification is difficult at sea-level and often involves a boat based spotter directing the collector. b—Close up of free swimming *C*.*fleckeri* (footage collected at ~1.5m from a 90degree angle with polarisation filter in 0.5m depth). c—Transect drone footage from Napranum with two *C*.*fleckeri* (indicated by red circle).
(MP4)

S1 Data (CSV)

S2 Data (CSV)

S3 Data (CSV)

S4 Data (CSV)

S1 Script (R)
