# Supplementary figures and images for: Bay watch: Using unmanned aerial vehicles (UAV’s) to survey the box jellyfish Chironex fleckeri
Source: PLoS One. 2020 Oct 29;15(10):e0241410. doi: 10.1371/journal.pone.0241410 (PMC7595306; doi:10.1371/journal.pone.0241410)

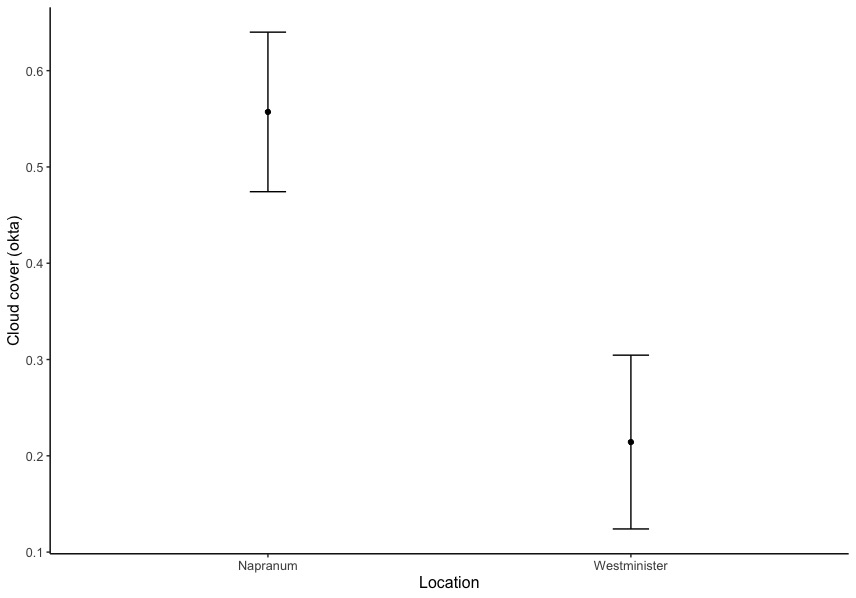

Supplement: S1 Fig — Napranum, Westminster (mean ±SE). (TIF) [file pone.0241410.s001.tif]
